# The effect of typicality training on costly safety behavior generalization

**DOI:** 10.1007/s00426-024-01979-0

**Published:** 2024-06-01

**Authors:** Işık E. Kesim, Andre Pittig, Alex H. K. Wong

**Affiliations:** 1 Department of Psychology, Educational Sciences, and Child Studies, Erasmus School of Social and Behavioural Sciences, Erasmus University Rotterdam, Burgemeester Oudlaan 50, Rotterdam, 3062 PA The Netherlands; 2 Translational Psychotherapy, Institute of Psychology, Georg-August-Universität Göttingen, Kurze-Geismar-Straße 1, 37073 Göttingen, Germany

## Abstract

**Background and objectives:**

Typicality asymmetry in generalization refers to enhanced fear generalization when trained with typical compared to atypical exemplars. Typical exemplars are highly representative of their category, whereas atypical exemplars are less representative. Individual risk factors, such as trait anxiety, attenuate this effect, due to the high level of threat ambiguity of atypical exemplars. Although recent research provided evidence for generalization of safety behavior, it is unclear whether this generalization also follows typicality asymmetry. This study examined (1) whether participants exhibited typicality asymmetry in the generalization of safety behavior and (2) whether this effect would be attenuated by individual risk factors, such as intolerance of uncertainty and trait anxiety.

**Methods:**

Participants were trained with either typical (Typical group, *n* = 53) or atypical (Atypical group, *n* = 55) exemplars in a fear and avoidance conditioning procedure. Participants acquired differential conditioned fear and costly safety behavior to the threat- and safety-related exemplars. In a following Generalization Test, the degree of safety behavior to novel exemplars of the same categories was tested.

**Results:**

The Atypical group showed greater differential safety behavior responses compared to the Typical group. Higher trait anxiety was associated with lower differential safety behavior generalization, driven by an increase in generalized responding to novel safety-related exemplars. *Limitations*: This study used hypothetical cost instead of real cost.

**Conclusions:**

Training with atypical exemplars led to greater safety behavior generalization. Moreover, individuals with high trait anxiety show impaired safety behavior generalization.

**Supplementary Information:**

The online version contains supplementary material available at 10.1007/s00426-024-01979-0.

## Introduction

Fear conditioning refers to the repeated pairing of an initially neutral conditioned stimulus (CS+) and an aversive unconditioned stimulus (US). Consequently, the CS + becomes a signal of threat and elicits conditioned fear responses such as elevated threat expectancy and skin conductance responses (SCRs). In many cases, fear conditioning was thought to model the development and maintenance of adaptive and maladaptive anxiety (Craske et al., 2018; Lissek et al., 2008; Mineka & Zinbarg, 2006).

Accordingly, research has utilized fear conditioning models to examine fear learning patterns in healthy individuals and individuals with anxiety-related disorders (Craske et al., 2018; Dunsmoor & Paz, 2015). A pattern linked to clinical anxiety is excessive fear generalization (Duits et al., 2015; Dunsmoor & Paz, 2015; Dymond et al., 2015). Fear generalization refers to fear acquired towards a CS + spreading to a range of novel stimuli that resemble the CS + even if they were never paired with the US (Dunsmoor et al., 2009). Recent research has demonstrated that fear can generalize to novel stimuli that are conceptually related to the CS+ (Bennett et al., 2015; Mertens et al., 2021; Wong & Lovibond, 2021). In categorical generalization, for example, fear generalized to novel stimuli that are categorically related to the CS + despite having no prior history with the US itself (e.g., Dunsmoor et al., 2012; Dunsmoor & Murphy, 2014; Lee et al., 2019; Wong & Pittig, 2020). In these studies, exemplars from one category (e.g., mammals; the threat category) were paired with an aversive US, whereas exemplars from another category (e.g., tools) were never reinforced (safe category). Later in the generalization test, novel exemplars of the two categories were presented. In general, participants exhibited stronger fear responses to novel exemplars of the threat category compared to those of the safety category (Dunsmoor et al., 2012; Dunsmoor & Murphy, 2014; Lee et al., 2019; Wong & Lovibond, 2020).

One factor that modulates the degree of categorical generalization is typicality (Dunsmoor & Murphy, 2014; Wong & Beckers, 2021). Typicality refers to the degree to which a stimulus represents the defining features of a given category. Dunsmoor and Murphy (2014) developed a categorical fear conditioning framework, where individuals were trained with either typical (i.e., exemplars that are great representatives of their category; cows as mammals) or atypical exemplars (i.e., exemplars that are less representative of their category; bats as mammals) and were tested with novel exemplars of reversed typicality. Individuals showed limited fear generalization to novel exemplars when trained with atypical compared to typical exemplars. This difference in fear generalization due to typicality is referred to as *typicality asymmetry*. According to the authors, training with typical exemplars likely facilitated the formation of category membership-US association, which might explain why novel exemplars of the same category yielded stronger fear generalization. Conversely, training with less representative exemplars of a category likely confined the acquisition of CS-US contingency to individual exemplars, thus limiting fear generalization.

Typicality asymmetry was, however, only examined in (passive) conditioned responses, but not in active responses such as safety behaviors. Safety behaviors refer to active behavioral responses that minimize an expected threat onset when confronting a feared stimulus/situation. Safety behaviors become maladaptive when they are used excessively in the absence of realistic threat, a hallmark feature in anxiety disorders (Craske, 1999; Dymond & Roche, 2009). In laboratory settings, safety behaviors are modeled as US-avoidance responses (e.g., Flores et al., 2018; 2020; Levita et al., 2012; Lovibond et al., 2009; Pittig, 2019) which regards executing a designated response (e.g., pressing a specific key) during CS + presentation to effectively reduce the chance of the US onset. However, these low-cost avoidance responses do not entirely capture the pathological qualities of safety behaviors observed in anxiety-related disorders, as pathological safety behaviors often bear a cost. For instance, patients engaging in elaborated ritual behaviors to prevent perceived threat, which can disrupt daily activities. To this end, recent studies have also introduced competing rewards to US-avoidance to render them costly (Pittig et al., 2021), allowing laboratory models to mirror pathological safety behaviors observed in anxiety-related disorders (Pittig et al., 2020). Therefore, the current study aimed to examine whether typicality asymmetry could be observed in costly US-avoidance.

There are other factors affecting the degree of fear generalization alongside typicality, for example, individual difference factors such as trait anxiety (Pittig et al., 2020; Wong & Lovibond, 2018) and intolerance of uncertainty (Morris et al., 2016, 2019). Trait anxiety and intolerance of uncertainty refers to individual’s dispositional tendency to experience anxiety in various situations even in the absence of a threat (Barlow, 2002) and to react negatively to uncertain and ambiguous situations (Buhr & Dugas, 2006; Carleton et al., 2007), respectively. Both trait anxiety and intolerance of uncertainty are regarded as risk factors in the development of anxiety disorders (Chambers et al., 2004; Gentes & Ruscio, 2011; Jorm et al., 2000; Shihata et al., 2016). Trait anxiety has been associated with various maladaptive fear learning patterns, and a stronger association between conditioned fear and avoidance behavior (see Pittig et al., 2014). For example, impaired safety learning was evident in trait anxious individuals consistently showing stronger self-reported distress to safety stimuli (see Gazendam et al., 2013). Similarly, intolerance of uncertainty was associated with incapability to discriminate between threatening and innocuous stimuli that are perceptually similar (Morriss et al., 2016), and with impaired extinction learning retention, reflected by individuals with high intolerance of uncertainty showing greater SCRs to an extinguished CS + after extinction learning compared to their low intolerance of uncertainty counterparts (see Morriss et al., 2016, 2019).

For categorical fear generalization, typicality asymmetry might be attenuated in trait-anxious individuals (Wong & Beckers, 2021). Training with atypical exemplars had likely confined learning to an individual exemplar – US association. In other words, participants trained with atypical exemplars were less likely to learn the threat predictiveness for the category (category membership – US association), but rather learned that individual exemplars were associated with the US (multiple CS – US associations). As a result, novel exemplars presented in a following generalization test became highly threat ambiguous as their threat predictiveness was relatively unclear. This high level of threat ambiguity represents a ‘weak’ situation, which is optimal to observe the maladaptive aspects of trait anxiety or intolerance of uncertainty on fear learning (Beckers et al., 2013). In line with this idea, typicality asymmetry was observed across all participants but was attenuated in high-trait anxious individuals compared to their low-trait counterparts (i.e., high-trait anxious individuals showing stronger fear generalization compared to their low-trait counterparts despite training with atypical exemplars).

The current study sought to examine whether individual risk factors attenuate typicality asymmetry in US-avoidance generalization. We followed the preliminary work of Wong and Beckers (2021) and expanded it to costly US-avoidance. In line with preliminary findings, we hypothesized that participants trained with typical exemplars would exhibit stronger US-avoidance generalization compared to those trained with atypical exemplars (i.e., typicality asymmetry). Secondly, we hypothesized that typicality asymmetry in generalization would be reduced in individuals high in trait anxiety or high in intolerance of uncertainty.

## Method

### Participants

A simulation-based power analysis was performed in R using mixed power package (Kumle et al., 2021). The estimated power was based on a previous study examining the effect of trait anxiety on typicality asymmetry in fear generalization (Wong & Beckers, 2021). According to the simulation-based power analyses, at least 100 participants were required to attain a power of 92.3% to detect an expected effect size of b = 1.69, or R2 = 0.02 (Jaeger et al., 2017) (see preregistration https://osf.io/d75rg). A total of 110 psychology undergraduates (17 men, 91 women, and 2 non-binary/other) from Erasmus University Rotterdam were recruited as participants and received mandatory course credits for their participation. Participants’ ages ranged from 18 to 30 (*M* = 20, and *SD* = 2). This study received ethics approval (ETH2122-0452) from the Research Ethics Review Committee at Erasmus University Rotterdam in accordance with the Declaration of Helsinki.

### Apparatus, stimuli, and materials

Twelve greyscale images from the mammal and bird categories (see Table 1) served as the category exemplars. They were previously rated for their typicality of membership on a Likert scale ranging from 1 to 7 (1 = not at all typical to 7 = highly typical; Wong & Beckers, 2021). The atypical exemplars had a mean typicality rating of 2.7 (*SD* = 1.6), whereas typical exemplars had a mean typicality rating of 6.6 (*SD* = 0.8). The intermediate exemplars had a mean rating of 4.7 (*SD* = 1.7).

Skin conductance was measured via two Ag/AgCl electrodes connected to a BioPac system at a sampling rate of 1000 Hz. The aversive noise US was a 500 ms noise blast at 100db administered via headphones connected to a sound amplifier.

Participants completed the English-translated Intolerance of Uncertainty Scale (IUS; Freestone et al., 1994; translated; Buhr, & Dugas, 2002) and the short version of Depression, Anxiety, and Stress Scale (DASS-21; Lovibond & Lovibond, 1995). IUS was used to assess whether individuals found uncertain situations to be distressing. IUS consisted of 27 items, rated on a Likert scale ranging from 1 to 5 (1 = *not at all characteristics of me* to 5 = *entirely characteristic of me*). The English-translated version of IUS yielded an excellent internal consistency (Cronbach’s *α* = 0.94) (Buhr, & Dugas, 2002). DASS-21 consisted of 21 items, rated on a 4-point Likert scale ranging from 0 to 3 (0 = *did not apply to me at all* to 3 = *applied to me very much or most of the time*). Furthermore, DASS-21 validly measures and discriminates between three negative emotional states (i.e., depression, anxiety, and stress) (Henry & Crawford, 2005). Moreover, the anxiety subscale had an excellent internal consistency (Cronbach’s *α* = 0.89) (Coker et al., 2018).

**Table 1:** Bird and mammal exemplars used in the current study

Typicality	Birds	Mammals
Typical	Hummingbird, Pigeon, Sparrow	Bear, Cow, Gorilla
Atypical	Cassowary, Emu, Penguin	Bat, Platypus, Seal
Intermediate	Duck, Flamingo, Kiwi, Peahen, Swan, Turkey	Alpaca, Camel, Dolphin, Otter, Rat, Sloth

### Procedure

The study began after participants provided informed consent. During the pre-experimental phase, headphones were placed on the participants which initiated the noise familiarization phase. Participants received 4 noise blasts with increasing intensity, starting from 30 dB to 100dB. The intensity of the final noise US was lowered to 95dB if participants found it too aversive. A reward-matching phase was carried out immediately after. Participants were presented with questions regarding whether they would tolerate the noise blast for a certain amount of hypothetical financial reward (“Are you willing to tolerate the noise when given €__?”) ranging between 5 and 31 cents in odd numbers (e.g., 5, 7, …,31 cents) in a randomized order. Participants responded verbally by saying YES or NO. Then the maximum competing reward was uniquely calculated for each participant as the amount between the highest hypothetical monetary reward that received a “NO” and the lowest hypothetical monetary reward that received a “YES”. For instance, if a participant agreed to tolerate the noise US for rewards ranging from 17 to 31 cents (i.e., responding “YES”) but refused to tolerate it (i.e., responding “NO”) for rewards ranging from 5 to 15 cents (i.e., responding “NO”), the maximum competing reward would be 16 cents. This individually calibrated level of reward was sufficient to create a conflict between avoidance and approach (see Schlund et al., 2016). Hereafter, participants were asked to fill in the IUS (Buhr, & Dugas, 2002) and the DASS-21 (Lovibond & Lovibond, 1995). The experimental phases started immediately after. The design of the current study is shown in Table 2.

**Table 2:** Design of the current study

Pavlovian fear acquisition	Costly US-avoidance acquisition	Generalization Test
CS1+ (9) CS2- (9)	CS1*[+, €] (9) CS2*- [€] (9)	GS1-* [€] (9) GS2-* [€] (9)

+ indicates US presentation; - indicates US omission; * indicates the availability of US-avoidance; [+] indicates the presentation of US and [€] indicates competing reward, the presentation of US and competing reward depend on US-avoidance; number in parentheses indicates the number of trials

#### Pavlovian fear acquisition

Participants were informed that various exemplars (CS) would be followed by a noise blast (US) and were prompted to learn the relation between the CSs and the aversive noise unconditioned stimulus (US). The Pavlovian fear acquisition phase had three blocks. In each block, three different mammal exemplars served as the CS + s whereas three different bird exemplars served as the CS-s, thus amounting to a total of 9 CS + trials and 9 CS- trials. The CSs were counterbalanced across participants. The CS presentation was pseudo-randomized, meaning that the same CSs were not presented more than twice in a row. The CS + s were fully reinforced by the noise US while the CS-s were never reinforced. Importantly, typical exemplars served as the CS + s in the Typical group whereas atypical exemplars served as the CS + s in the Atypical group. In both groups, exemplars of intermediate typicality served as the CS-s (see Table 1). All CSs were presented on screen for 8 s with the US-expectancy scale. Participants were prompted to indicate their US-expectancy on each trial. Hence, participants were presented with a visual analog scale (VAS) below the CSs ranging from 0 to 100% with a 1% increment (0% = *Certainly NO noise* to 100% = *Certain noise*). The US-expectancy scale disappeared with the CS offset and the noise US was delivered immediately after the CS + offset. The inter-trial intervals ranged from 15 to 18 s in all three phases.

#### Costly US-avoidance acquisition

At the start of the Costly US-avoidance acquisition phase, participants were informed about their opportunity to avoid the potential noise US by indicating their avoidance responses on the US-avoidance scale (Wong & Pittig, 2021). Therefore, a VAS ranging from 0 to 100% with 1% increments was presented first below the CSs. The US-avoidance scale was negatively proportional to the chance of US onset and the maximum reward. For instance, if a participant selected 60% on the US-avoidance scale, there would be a 40% chance of receiving the noise US after CS + offset. However, participants would only receive 40% of the maximum reward. The costly US-avoidance acquisition phase consisted of three blocks. Each block consisted of the three CS + s and three CS-s presented in the previous phase. After participants indicated their US-avoidance responses, the CS and the US-avoidance scale disappeared simultaneously. Following a 1 s fixation cross, the same CS was presented for 8 s with the US-expectancy scale below. Participants were prompted to indicate their US-expectancy responses. After CS offset, a US might be administered depending on the US-avoidance response and CS type, followed by a 2 s reward feedback.

#### Generalization test

This phase followed seamlessly from the same block and trial structure as the previous phase. Three novel stimuli from the CS + category (GS+) and three novel stimuli from the CS- category (GS-) were presented once in each of the three blocks. The Atypical group was presented with typical GS + whereas the Typical group was presented with atypical GS+. Both groups received GS- of intermediate typicality. On each trial, participants were presented with GSs and were prompted to indicate their US-avoidance responses. After a US-avoidance response was made, the GS and US-avoidance scale disappeared followed by a 1 s fixation cross. The same GSs then reappeared for 8 s with a US-expectancy scale. A 2 s reward feedback followed the GS offset. Importantly, neither the GS + nor GS- was reinforced, and participants were not given this information prior.

#### Scoring and analysis

Skin conductance was measured throughout the experiment. Only SCRs 1s after CS/GS onset to CS/GS offset were included for analysis. To remove the high-frequency noise from the skin conductance data, we applied a 1 Hz low-pass filter and a 50 Hz notch filter. After, SCRs were calculated by taking the difference between the maximum response and the preceding trough (Pineles et al., 2009). SCRs were then square-rooted to reduce skewness (Boucsein et al., 2012).

All data were analyzed within a linear mixed model framework in R with lmer package (Bates et al., 2012). All planned analyses were pre-registered on OSF (https://osf.io/bj658).

We carried out two separate manipulation checks. First, we analyzed whether participants had higher expectancy ratings and greater SCRs to the CS + than the CS- during Pavlovian fear acquisition. Expectancy ratings and SCRs were used as the dependent variable in two separate models. CS type (CS + vs. CS-), Block (linear trend repeated measure across blocks), and their interaction, served as fixed effects in both models. These models captured whether fear learning to the CS + was acquired. Group (Typical vs. Atypical) and trait anxiety/intolerance of uncertainty (as continuous variables) were subsequently added to these models as fixed effects to see if these factors had any effect on fear learning. Second, we analyzed whether participants had acquired stronger US-avoidance to CS + compared to CS- during Costly US-avoidance acquisition. Accordingly, US-avoidance responses served as the dependent variable, while CS type, Block, and their interaction served as fixed effects. Group and trait anxiety/intolerance of uncertainty were subsequently added to these models to examine whether these factors had any effect on costly US-avoidance acquisition.

Regarding the main hypotheses, we investigated whether typicality asymmetry in US-avoidance generalization was observed. Specifically, we examined whether the Typical group showed greater differential costly US-avoidance to the GSs compared to the Atypical group during the Generalization test phase. To this end, we employed a model where US-avoidance responses served as the dependent variable. Stimulus type (GS + vs. GS-), Group, Block, and their interactions served as fixed effects. Then, we tested whether higher trait anxiety or intolerance of uncertainty reduced typicality asymmetry in US-avoidance generalization (i.e., higher trait anxiety/intolerance of uncertainty indexes greater differential responding to the GSs in the Atypical group). Therefore, US-avoidance responses during the Generalization test phase served as the dependent variable. Stimulus type, Group, Block, and trait anxiety/intolerance of uncertainty, and their interactions served as fixed effects.

Finally, we implemented two separate models that only included the first block of Generalization test to minimize confounding extinction learning (i.e., to minimize the confounding reduction in responses as all GSs were not reinforced in Generalization test). US-avoidance served as the dependent variable in both models. The first model included Stimulus type, Group, and their interaction as fixed effects. In the second model, Stimulus type, Group, trait anxiety/intolerance of uncertainty, and their interactions served as the fixed effects. Participants served as the random effect for all the aforementioned linear mixed models.

The degree of significance was reported with Satterthwaite approximation for degrees of freedom in all models (Satterthwaite, 1941). Finally, we expected that Group and trait anxiety/intolerance of uncertainty would have no effect on differential US-expectancy and costly US-avoidance acquisition. Therefore, we used the Bayes Model to confirm the absence of an effect (Kruschke, 2015). We obtained 95% highest density intervals (HDI), which contain the most credible values. Then, we looked at the posterior distribution that fell under the range of area around the null value, also referred to as the Region of Practical Equivalence (ROPE) (Kruschke, 2015). We then calculated the percentage of HDIs that fell under ROPE (Kruschke, 2015; Kruschke & Liddell, 2018).

## Results

As preregistered, statistical analyses were restricted to participants who had demonstrated differential fear conditioning and CS-US contingency awareness. In other words, only participants who had higher averaged US-expectancy ratings for the CS + compared to the CS- in the last *Pavlovian fear acquisition* block (i.e., the last 3 trials of CS + and the last 3 trials of CS-) were included to the statistical analyses. A total of 2 participants were excluded from this study, leaving 108 participants (53 in the Typical and 55 in the Atypical group).

### Pavlovian fear acquisition phase

**Fig. 1:**
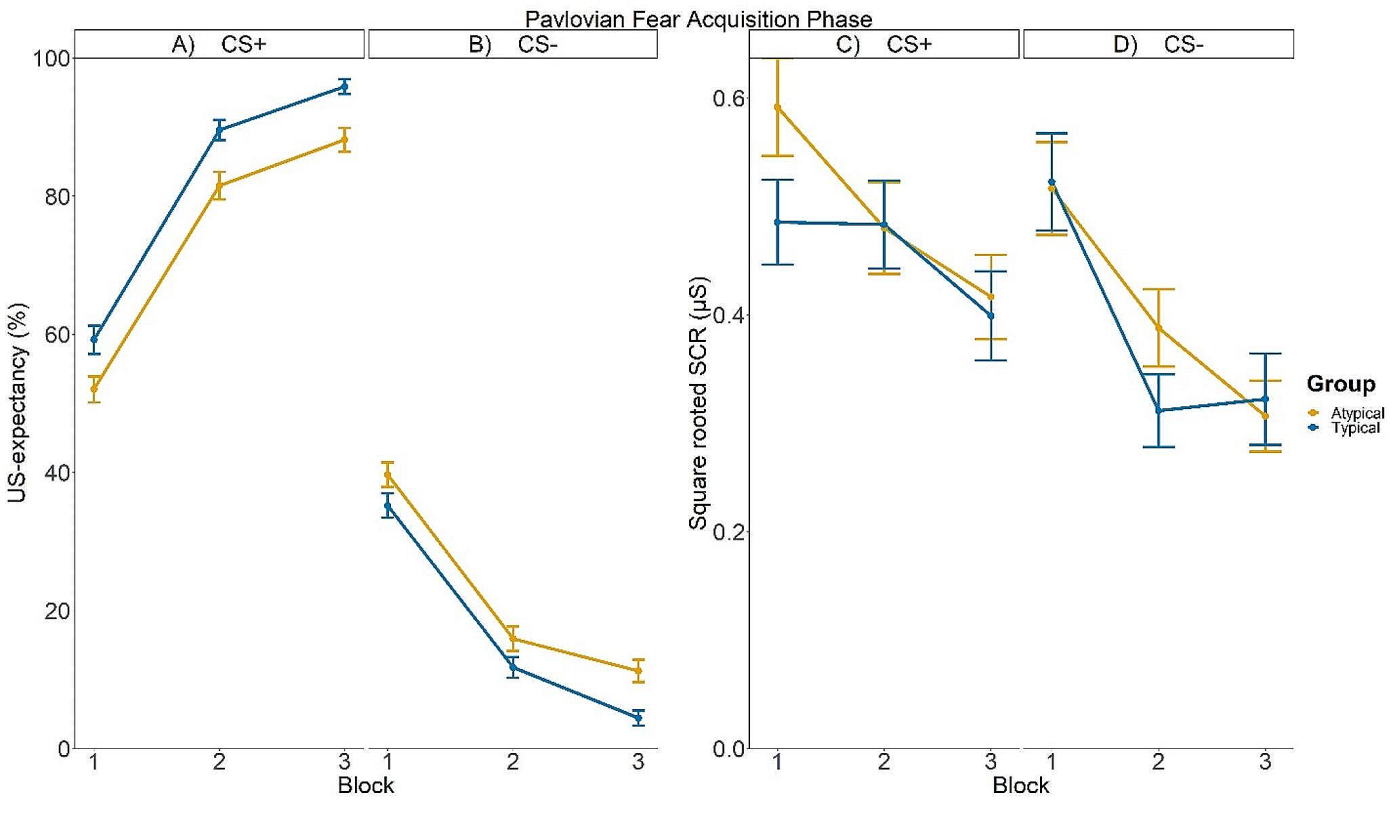
Left panel: Mean US-expectancy ratings to the CS+ (**A**) and the CS- (**B**) across Pavlovian fear acquisition blocks. Right Panel: Mean square root SCRs to the CS+ (**C**) and the CS- (**D**) across Pavlovian fear acquisition blocks. The orange and blue bars indicate responding in the Typical group and the Atypical group, respectively. Error bars indicate the standard error of the mean

Figure 1A-B show the mean US-expectancy ratings across Pavlovian fear acquisition blocks in each typicality group. Averaged over typicality groups, participants developed higher US expectancy ratings to the CS + compared to the CS- across acquisition block, whereas this difference increased across blocks. This was supported by a significant interaction between CS type and Block averaged across Group (*b*CStype×Block = -1187.22, *SE* = 43.61, *p* < .001). Unexpectedly, the interaction between Group and CS type averaged over Block was significant (*b*Group×CStype = -12.79, *SE* = 2.32, *p* < .001). This suggests that the Typical group had greater US-expectancy ratings to CS + compared to CS- averaged across the *Pavlovian fear acquisition* blocks when compared to the Atypical group. No other interactions involving Group reached significance (smallest *p* = .545).

No interactions involving intolerance of intolerance of uncertainty reached significance (smallest *p* = .194). The Bayesian model confirmed the absence of the effect of intolerance of uncertainty on the differential US-expectancy responses averaged across the *Pavlovian fear acquisition* blocks, as 100% of the HDIs depicting the interaction between CS type, Group and intolerance of uncertainty fell within ROPE.

On the other hand, an increase in trait anxiety was associated with impaired differential US-expectancy ratings to the CSs averaged over Block and Group (*b*CStype×TA = 0.48, *SE* = 0.17, *p* = .005). No other interactions involving trait anxiety reached significance (smallest *p* = .071).

Figure 1C-D show the square root SCRs across Pavlovian fear acquisition blocks in each group. Participants had stronger SCRs to the CS + s compared to the CS-s averaged over blocks and groups. This was supported by a significant main effect of CS type (*b*CStype = -0.08, *SE* = 0.01, *p* < .001). None of the interactions involving Group reached significance (smallest *p* = .162), suggesting there was no evidence that the two groups differed in SCRs during Pavlovian fear acquisition. Additionally, no interactions involving trait anxiety/intolerance of uncertainty reached significance (smallest *p* = .132). The Bayesian model confirmed the absence of these effects, as 100% of the HDIs depicting the interactions involving CS type, Group and trait anxiety/intolerance of uncertainty fell within ROPE.

### Costly US-avoidance acquisition phase

**Fig. 2:**
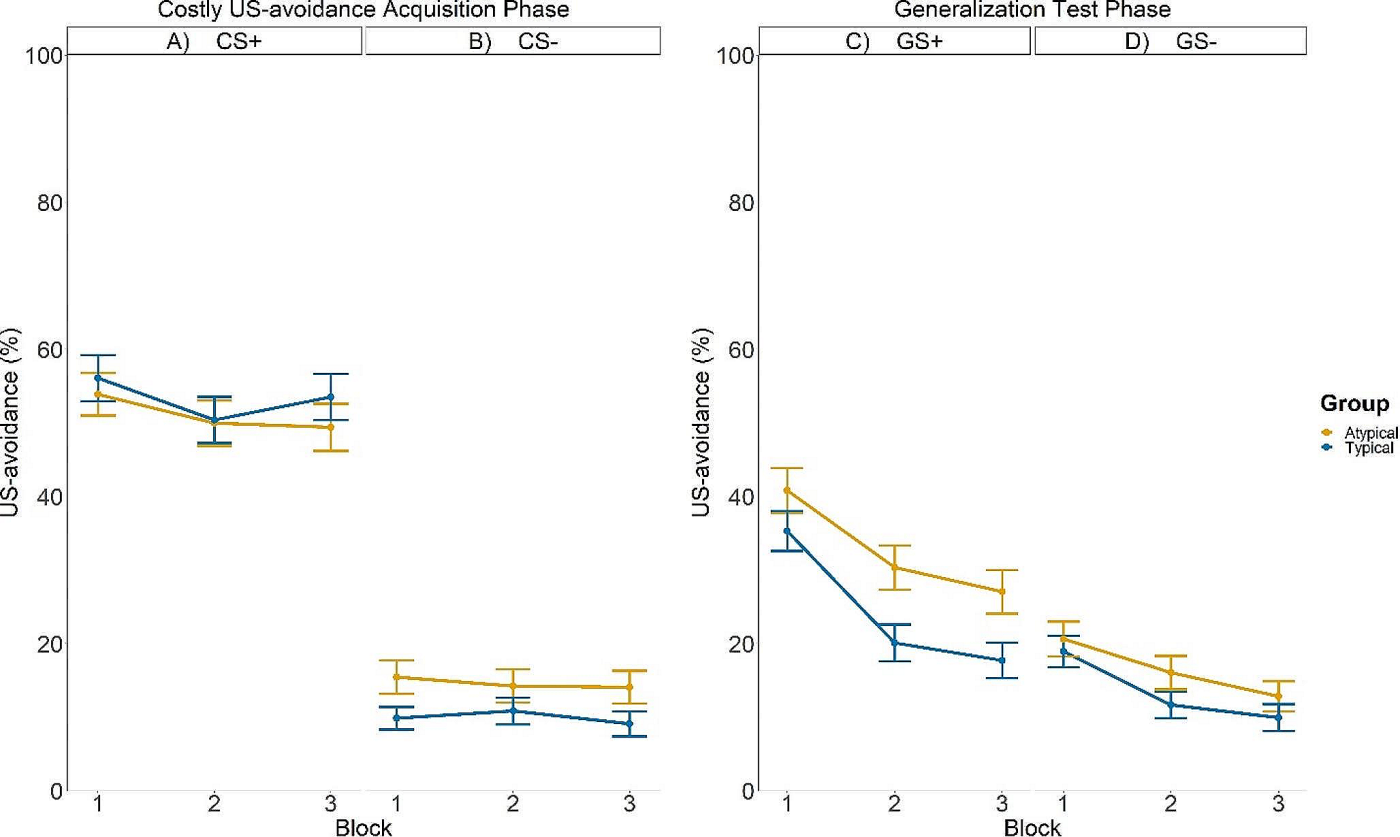
Left panel: US-avoidance to the CS+ (**A**) and the CS- (**B**) across Costly US-avoidance acquisition blocks. Right Panel: US-avoidance to the GS+ (**C**) and the GS- (**D**) across Generalization Test blocks. GS + indicates novel categorical exemplars that belong with CS+; GS- indicates novel categorical exemplars that belong with CSs. The orange and blue bars indicate responding in the Typical group and the Atypical group, respectively. Error bars indicate the standard error of the mean

Figure 2A-B show the mean US-avoidance responses across Costly US-avoidance acquisition blocks in each group. Averaged across Block and Group, US-avoidance for the CS + exemplars were higher compared to the CS- exemplars (*b*CStype= -39.88, *SE* = 1.11, *p* < .001), indicating differential US-avoidance to the CSs during the *Costly US-avoidance acquisition phase.* Unexpectedly, the Typical group had greater differential US-avoidance responses to the CSs compared to the Atypical group, *b*CStype×Group = -6.90, *SE* = 2.21, *p* = .002. No other interactions involving the Group had reached significance (smallest *p* = .725).

Furthermore, no interaction involving intolerance of uncertainty reached significance (smallest *p* = .161). The Bayesian model confirmed the absence of the effect of intolerance of uncertainty on the differential US-avoidance responses, as 100% of the HDIs depicting the interactions involving CS type, Group, and intolerance of uncertainty fell within ROPE. The three-way interaction involving Group, CS type, and trait anxiety reached significance (*b*CStype×Group×TA= -1.52, *SE* = 0.33, *p* < .001). This suggests that increased trait anxiety was associated with decreased differential US-avoidance responses to the CSs; this pattern was stronger in the Atypical group compared to the Typical group averaged across *Costly US-avoidance acquisition* blocks. US-expectancy ratings and SCRs were also assessed after US-avoidance responses were made. The analyses of US expectancy and SCRs were reported in the Supplementary Materials.

### Generalization test

Figure 2C-D illustrate the mean US-avoidance across *Generalization test* blocks in each group. We observed that averaged across Groups, participants showed greater costly US-avoidance to the GS + compared to the GS-, while this difference decreased across blocks (*b*Stimulustype×Block = 130.66, *SE* = 42.64, *p* = .002). Unexpectedly, the Atypical group had stronger differential US-avoidance generalization, evidenced by a greater difference in responding to the GS + compared to the GS- than the Typical group when averaging across blocks (*b*Stimulustype×Group = 5.37, *SE* = 1.93, *p* = .007). This suggests the expected typicality asymmetry in US-avoidance generalization (stronger generalization in the Typical group than the Atypical group) went into an opposite direction. No other effects involving Group reached significance (smallest *p* = .578). See Supplementary Materials for the US-expectancy ratings and SCRs analyses.

**Fig. 3:**
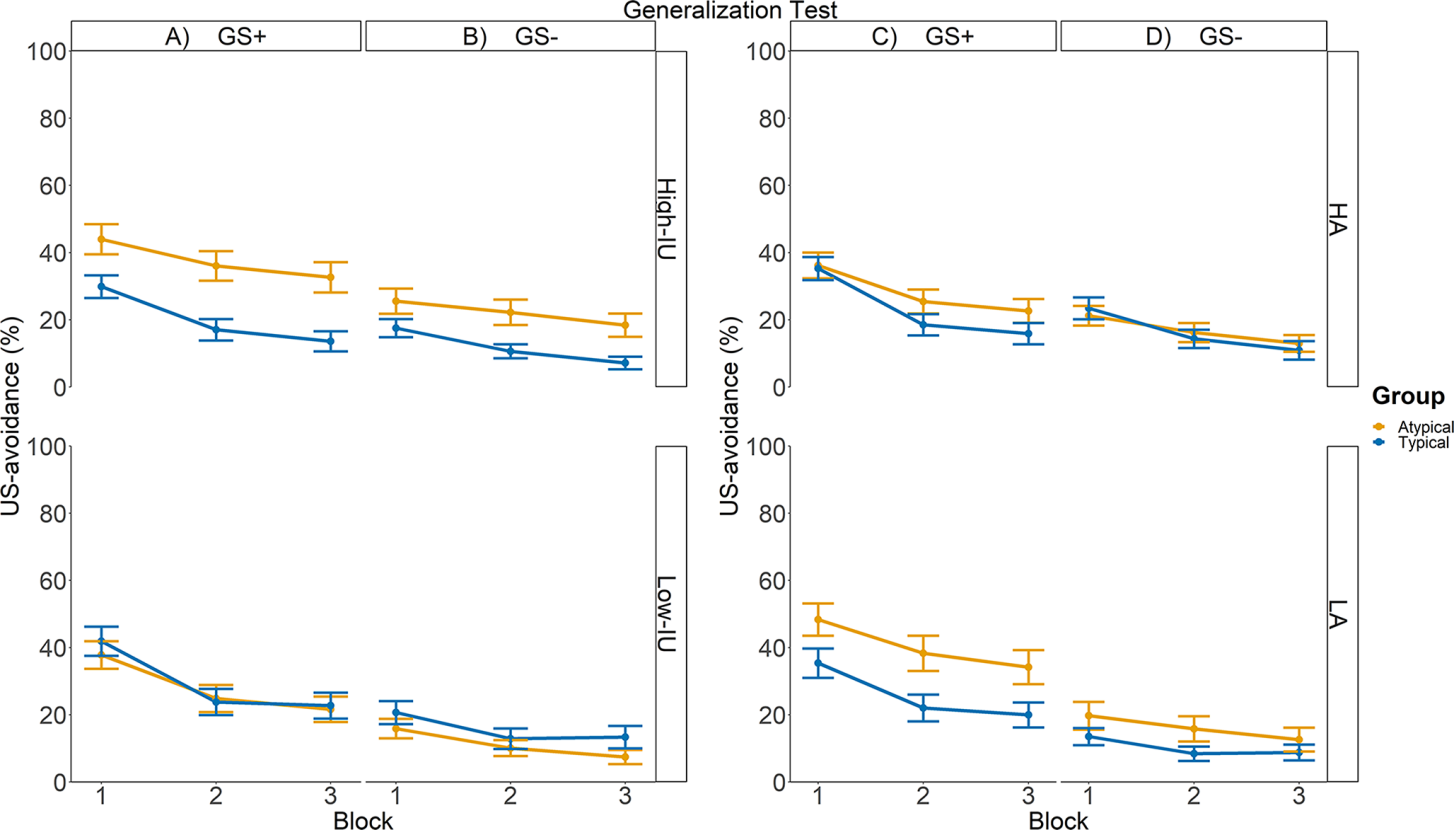
A median split was performed to categorize high and low trait anxiety/intolerance of uncertainty individuals. This was done for better visualization of the data. Left Panel: Mean US-avoidance responses of High- and Low- intolerance of uncertainty participants across Generalization Test blocks. Right Panel: Mean US-avoidance responses of High- and Low- trait anxious participants across Generalization Test blocks. GS + indicates novel categorical exemplars that belong with CS+; GS- indicates novel categorical exemplars that belong with CSs. The orange and blue bars indicate responding in the Typical group and the Atypical group, respectively. Error bars indicate the standard error of the mean

Figure 3A-D show the mean US-avoidance across *Generalization Test* blocks for high and low trait anxiety/intolerance of uncertainty participants between typicality groups respectively. No interactions involving intolerance of uncertainty reached significance (smallest *p* = .594). This suggests that there was no evidence that an increase in intolerance of uncertainty was associated with different degrees of US-avoidance generalization. Regarding trait anxiety, averaged over Group and Block, an increase in trait anxiety was associated with a decrease in differential US-avoidance to the GSs, supported by a significant interaction between Stimulus type and trait anxiety (*b*Stimulustype×TA = 0.55, *SE* = 0.15, *p* < .001). Follow-up robust regression analyses showed that an increase in trait anxiety was associated with an increase in generalized US-avoidance responses to the GS-, *β*TA = 0.0093, SE = 0.0017, *p* < .001, but there was no evidence that trait anxiety was associated with generalized US-avoidance responses to the GS+, *β*TA = -0.0011, SE = 0.0050, *p* = .829. No other interactions involving trait anxiety reached significance (smallest *p* = .478).

### First block of generalization test

To minimize confounding extinction learning, we examined only the first block of the *Generalization Test*. US-avoidance responses were generalized selectively to the GS + in both groups, supported by a main effect of Stimulus type (*b*StimulusType = -18.32, *SE* = 1.74, *p* < .001). However, there was no evidence for any group differences in costly US-avoidance generalization, *b*StimulusType×Group = 3.81, *SE* = 3.48, *p* = .275, suggesting no evidence for typicality asymmetry in US-avoidance generalization. Moreover, no interaction involving trait anxiety/intolerance of uncertainty reached significance, (smallest *p* = .169). This suggests that there was no evidence that trait anxiety/intolerance of uncertainty had an effect on US-avoidance generalization in the first block of the *Generalization Test* phase.

## Discussion

The current study sought to extend typicality asymmetry in generalization of costly US-avoidance. Furthermore, we examined whether trait anxiety or intolerance of uncertainty would reduce typicality asymmetry in costly US-avoidance generalization. We expected participants trained with atypical exemplars to exhibit limited US-avoidance generalization compared to participants trained with typical exemplars. Moreover, we expected that this pattern would be weaker as trait anxiety or intolerance of uncertainty increased.

During Pavlovian fear acquisition, the Typical group had stronger differential US-expectancy ratings compared to the Atypical group, as further characterized by the Typical group showing higher US expectancies to the CS + and lower US expectancies to the CS- compared to the Atypical group. This difference was presumably due to the faster attribution of US predictiveness to the category membership in the Typical group. In contrast, the Atypical group likely attributed US occurrence to individual exemplars instead of to the category membership, thus leading to a delay in acquiring differential US-expectancy ratings (cf. Dunsmoor et al., 2012). Moreover, an increase in trait anxiety was associated with impaired differential costly US-avoidance responses, specifically in the Atypical group. This aligned with findings that trait anxiety is associated with impaired differential safety behaviors learning (Wake et al., 2021). This pattern might have only been observed in the Atypical group due to an increase in threat ambiguity, as the effects of trait anxiety on fear and avoidance learning are more likely to manifest under threat ambiguity (Beckers et al., 2013).

During the Generalization test, we observed stronger US-avoidance responses to the GS + s compared to GS-s, suggesting US-avoidance responses more selectively generalized to novel exemplars that shared category membership with the trained CS + category (Arnaudova et al., 2017; Dymond et al., 2014). This was in line with research that examined higher-order conceptual generalization of avoidance to novel stimuli that shared category membership or had semantic relation to the CS+ (Boyle et al., 2016; Dymond et al., 2011, 2014; Kloos et al., 2022). In contrast to our expectation, the Atypical group showed stronger differential costly US-avoidance to the GSs compared to the Typical group. This finding contradicted previous studies that found typicality asymmetry in fear generalization (Dunsmoor & Murphy, 2014; Wong & Beckers, 2021). However, this unexpected pattern was only observed across the entire Generalization test phase where extinction learning had already occurred, but not in the first test block when the effect of extinction learning was minimized. This suggests that the reversed typicality asymmetry effect was frail and was mostly driven by slow extinction learning to the GS + s. One possible reason for this is that the groups might have adopted different learning strategies during training. Due to the clear categorical membership of the training exemplars, the Typical group was likely to acquire both CS + category – US association (e.g., mammal exemplars predict shock) and CS- category – no US association (e.g., bird exemplars predict no shock). In contrast, the Atypical group received atypical CS + exemplars that were not representative of their category, thus more likely to have only acquired a CS- category – no US association. Acquiring only this association might have promoted the use of a learning strategy that all exemplars not belonging to the CS- category predict shock. The use of this strategy speculatively led to a state of hypervigilance, as there was no learnt CS + category – US association (e.g., only mammals predict shock) to confine generalized responding to one particular category. Hypervigilant of threat is thought to slow down extinction learning (see Armstrong et al., 2022).

Another possible explanation for the apparent slow extinction in the Atypical group is the learning of a CS + category only until Generalization test. During Generalization test, Atypical group received highly typical GS + exemplars, resulting in participants realizing that the GS + and CS + exemplars belong to the same category, thus retrospectively forming a CS + category – US association. Having merely acquired this association during Generalization test might lead to slower extinction learning.

Moreover, the current study sought to examine whether trait anxiety or intolerance of uncertainty would attenuate typicality asymmetry in US-avoidance generalization. We hypothesized that an increase in these risk factors would be associated with reduced typicality asymmetry. However, the current findings were not able to support this hypothesis, as no typicality asymmetry in US-avoidance generalization was found in the first place.

Averaged across group manipulation, an increase in trait anxiety was associated with impaired discriminative generalized US-avoidance to the GSs during the Generalization test (i.e., differential US-avoidance generalization was reduced as trait anxiety increased). This pattern was driven by trait anxious individuals exhibiting stronger generalized US-avoidance to the GS-. This pattern expands on findings that trait anxiety is associated with impaired safety learning (e.g., Baas et al., 2008; Chan & Lovibond, 1996) to generalized responding to novel safety stimuli.

On the other hand, we did not find any effects of intolerance of uncertainty of safety behaviors generalization, in contrast to findings that suggest intolerance of uncertainty is associated with stronger US-avoidance generalization (San Martin et al., 2020). The field of examining individual risk factors and avoidance learning is, however, still in its infancy (Wong et al., 2023). Therefore, it is important for future study to test the robustness of the effects of risk factors on avoidance learning (and its generalization) via replication.

One limitation of this study was the use of hypothetical reward. One may argue that using hypothetical reward may not model costly US-avoidance as participants may not view it as costly as a real reward. However, studies have used hypothetical reward and successfully reduced behavioral avoidance (e.g., Dibbets & Fonteyne, 2015; Pittig et al., 2014), suggesting that participants did pursue hypothetical rewards by not using avoidance responses. Furthermore, studies have shown that both hypothetical and reward rewards had similar effects on decision making (e.g., Jenkinson et al., 2008; Locey et al., 2011). Another limitation was the overlapping perceptual similarities between the two categories. For instance, both birds and bats have wings while both birds and platypuses have beaks. These shared perceptual features between exemplars in different categories could have confounded with categorical generalization.

## Conclusion

In conclusion, the current study replicated research regarding higher-order categorical safety behavior generalization (Dymond et al., 2011, 2014; Kloos et al., 2022). More specifically, we observed that participants successfully generalized from their training exemplars to the novel generalization exemplars. However, we did not replicate typicality asymmetry in costly safety behavior generalization. In fact, the Atypical group showed greater differential costly US-avoidance generalization compared to the Typical group, although this effect was most likely driven by the Atypical group exhibiting slower extinction learning to the GS + s. We have made two speculations in the failure to replicate this pattern, including the possible adaptation of a different learning strategies and the Atypical group learning on a categorical level only during Generalization test. Nevertheless, the findings showed that increased trait anxiety was associated with stronger generalized safety behaviors to exemplars that belong to the safety category, highlighting the maladaptive safety behaviors generalization to other innocuous situations observed in anxious individuals. It is worth noting that there is currently a lack of research examining the relationship between conceptual safety behavior generalization and individual risk factors. As such, this study makes an important contribution to the field by shedding light on the potential influence of individual risk factors on the maladaptive pathological avoidance observed in anxiety-related disorders.

### Electronic supplementary material

Below is the link to the electronic supplementary material.

Supplementary Material 1

## Data Availability

Data generated from this study are posted on Open Science Framework via https://osf.io/axc3p/.
