# Supplementary material for: The effect of typicality training on costly safety behavior generalization
Source: Psychol Res. 2024 Jun 1;88(5):1771–82. doi: 10.1007/s00426-024-01979-0 (PMC11281986; doi:10.1007/s00426-024-01979-0)
Supplement: Supplementary file 1 — Supplementary Material 1 [file 426_2024_1979_MOESM1_ESM.docx]

**Supplementary Materials**

1. ***US-expectancy ratings and SCRs graphs grouped by participants high and low in trait anxiety and intolerance of uncertainty***


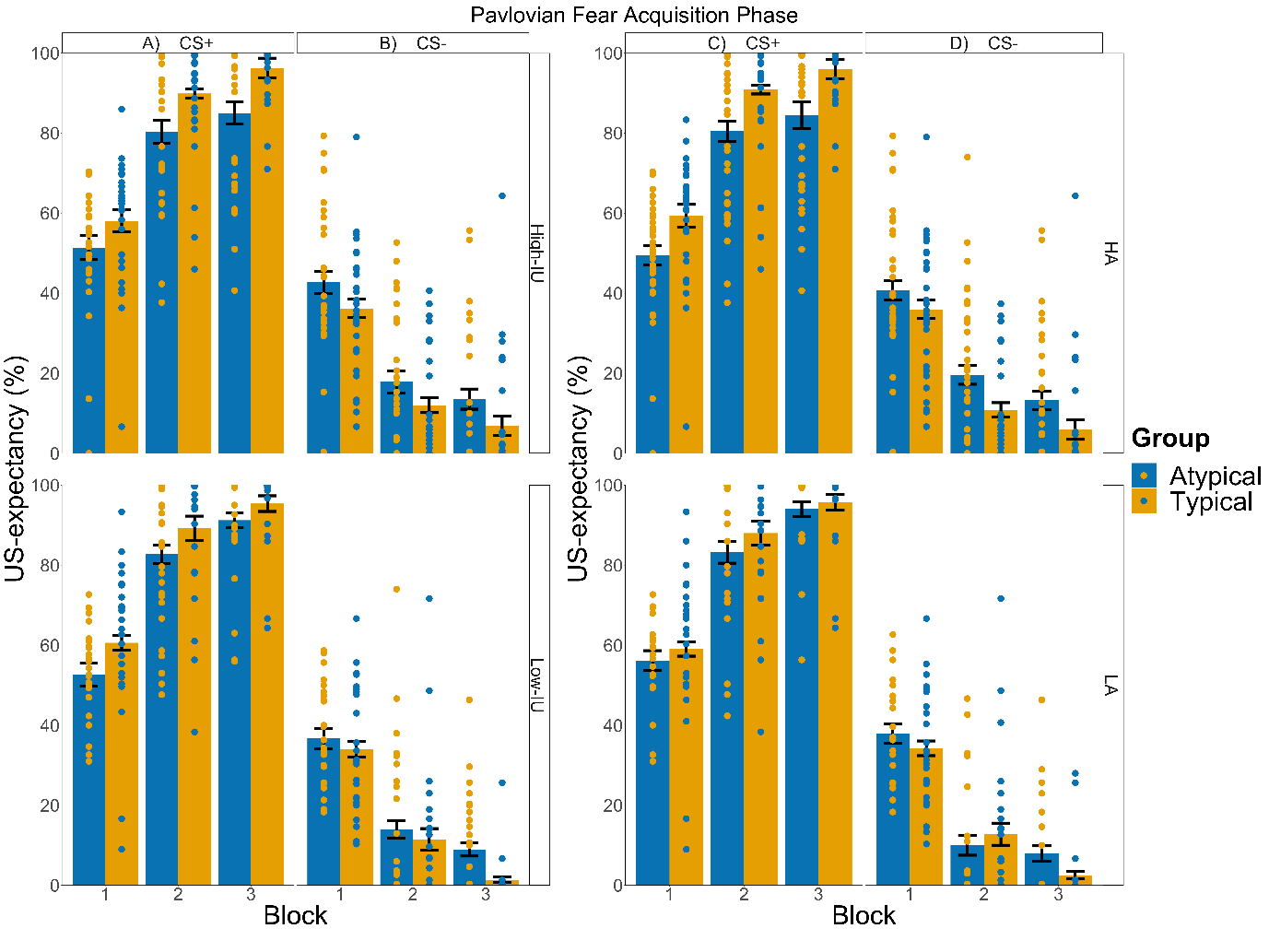

Fig. S1. A median split was performed to categorize high and low trait anxiety/intolerance of uncertainty individuals. This was done for better visualization of the data. Left Panel: Mean US-expectancy ratings of High- and Low- intolerance of uncertainty participants across Pavlovian fear acquisition blocks. Right Panel: Mean US-expectancy ratings of High- and Low- trait anxious participants across Pavlovian fear acquisition blocks. The orange and blue bars indicate responding in the Typical group and the Atypical group, respectively. Error bars indicate the standard error of the mean.


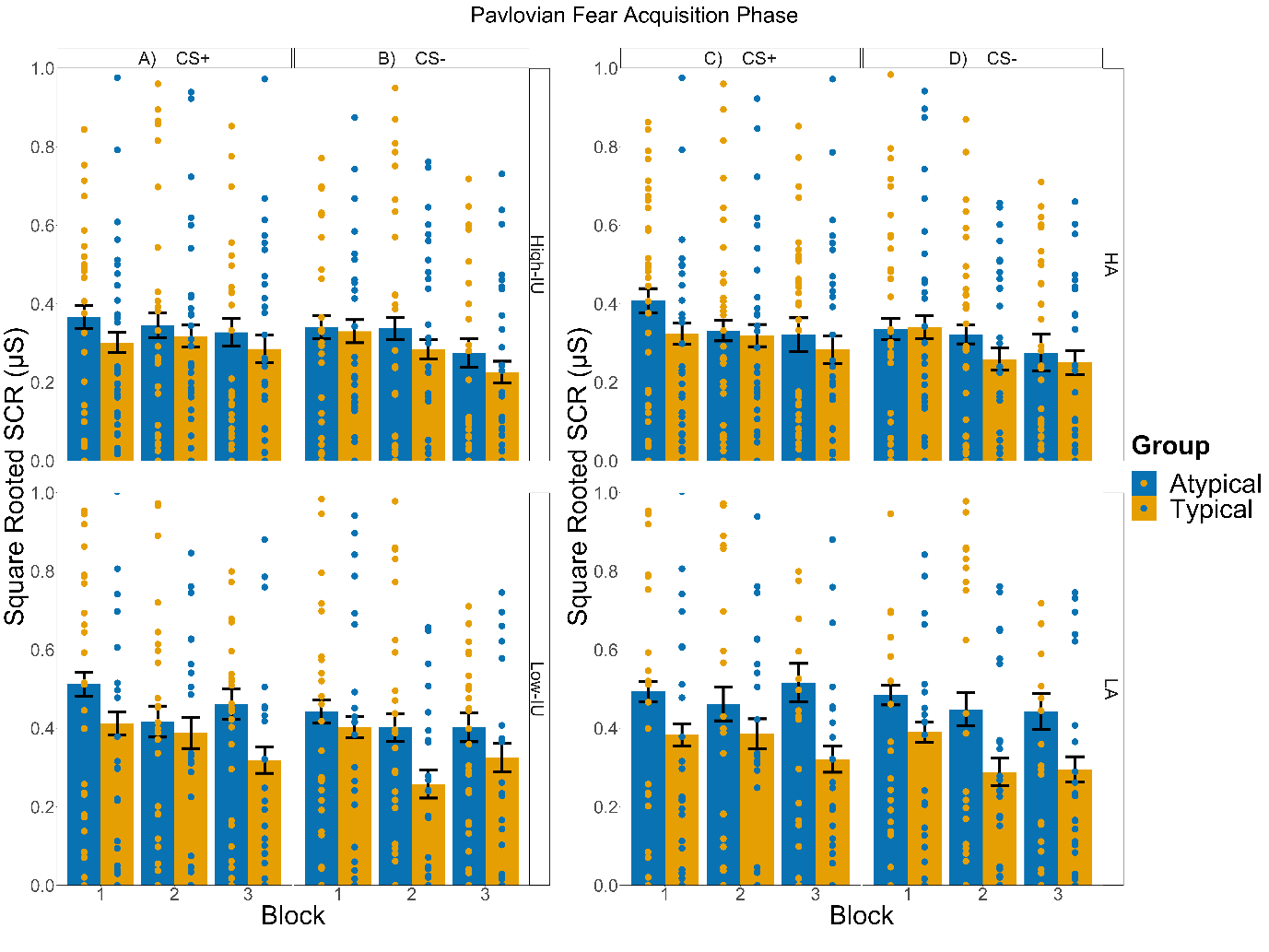

Fig. S2. A median split was performed to categorize high and low trait anxiety/intolerance of uncertainty individuals. This was done for better visualization of the data. Left Panel: Mean SCRs of High- and Low- intolerance of uncertainty participants across Pavlovian fear acquisition blocks. Right Panel: Mean SCRs of High- and Low- trait anxious participants across Pavlovian fear acquisition blocks. The orange and blue bars indicate responding in the Typical group and the Atypical group, respectively. Error bars indicate the standard error of the mean.

1. ***US expectancy ratings during Costly US-avoidance acquisition and Generalization test***

*Costly US-avoidance acquisition:*

Figure S1 shows the mean US-expectancy responses across Costly US-avoidance acquisition blocks in each group. A two-way interaction between CS type and Group reached significance (*b*CS type × Group = -7.81, *SE* = 2.28, *p* < .001). This suggest that the Typical group had greater US-expectancy ratings to CS+ compared to CS- averaged across the *Costly US-avoidance acquisition* when compared to the Atypical group. No interaction involving Blocks had reached significance (smallest *p* = 0.149).

Furthermore, the three-way interaction involving Group, CS type, and intolerance of uncertainty had reached significance, (*b*CStype × Group × IU= 0.81 *SE* = 0.12, *p* < .001). This suggest that an increase in intolerance of uncertainty was associated with decreased differential US-expectancy responding averaged across *Costly US-avoidance acquisition* blocks, specifically in the Typical group (see Fig. S2). Likewise, an increase in trait anxiety was associated with decreased differential US-expectancy responses, and this pattern was stronger in the Atypical group compared to the Typical group averaged across *Costly US-avoidance acquisition* blocks (see Fig. S2) This was supported by a significant three-way interaction *b*CStype × Group × TA= 1.46, *SE* = 0.33, *p* < .001 .

*Generalization Test:*

Participants had stronger US-expectancy responses to the CS+s compared to the CS-s averaged over blocks and groups. This was supported by a significant main effect of CS type (*b*CStype = -15.76, *SE* = 1.18, *p* < .001). Averaged across Blocks, the Atypical group had stronger differential US-expectancy generalization evidenced by the stronger responses to the GS+ compared to the GS- than the Typical group (*b*Stimulustype × Group= 6.76, *SE* =1.18, *p* < .001). This suggests that the Atypical group had stronger US-expectancy responses to novel generalization exemplars compared to the Typical group. No other effect reached significance (smallest *p* = .161).

No interactions involving IU reached significance (smallest *p* = .188). This suggests that there was no evidence that an increase in IU was associated with different degrees of US-expectancy generalization. With regard to trait anxiety, averaged over Group and Block, an increase in trait anxiety was associated with a decrease in differential US-expectancy to the GSs, supported by a significant interaction between Stimulus type and trait anxiety (*b*Stimulustype × TA = 0.55, *SE* = 0.17, *p* = .001). No other interactions involving trait anxiety reached significance (smallest *p* = .437).


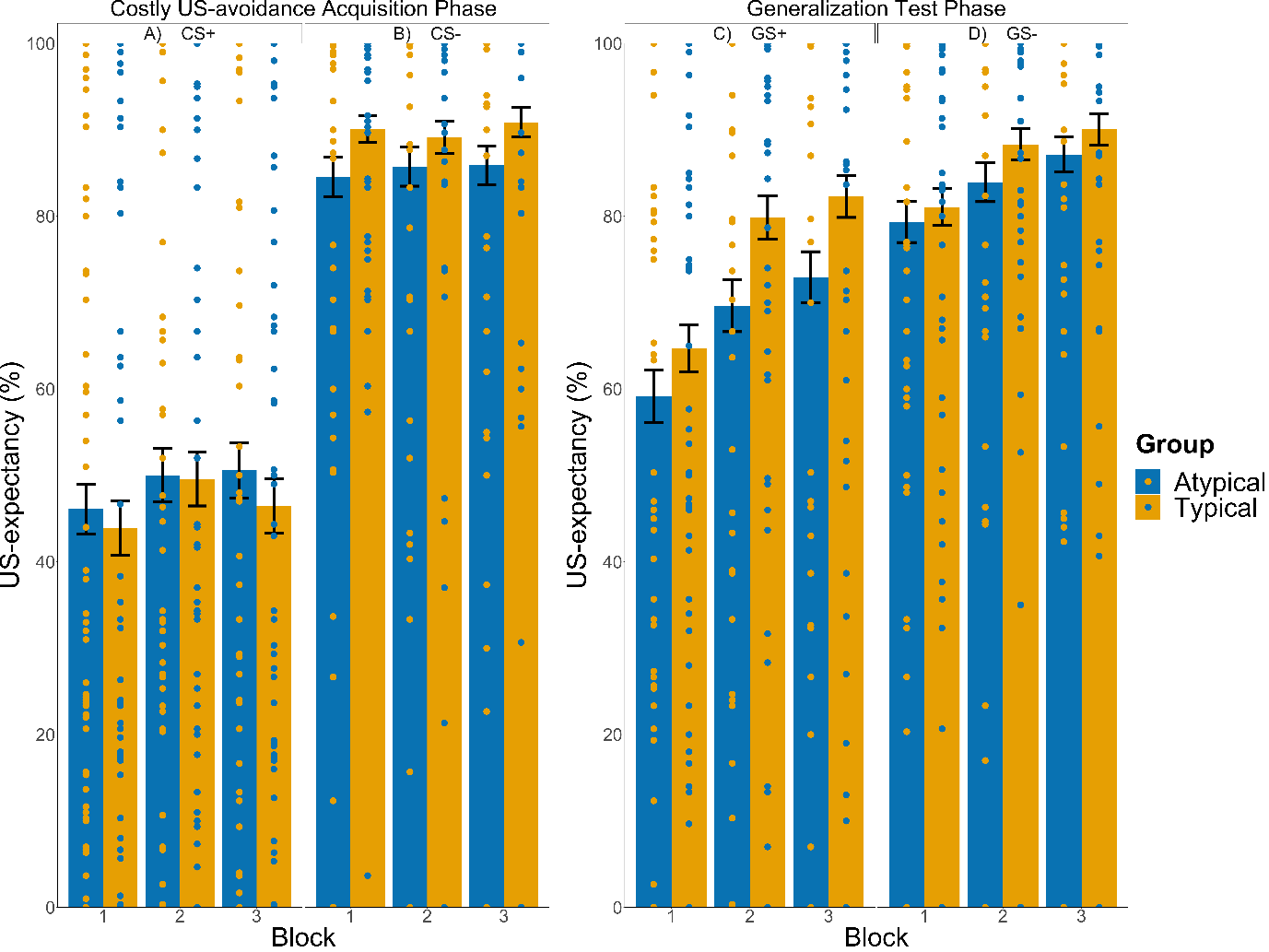


Fig. S3. Left panel: Mean US-expectancy ratings across Costly US-avoidance acquisition blocks for CS+ (A) and CS- (B).

Right Panel: Mean US-expectancy ratings across Generalization test blocks. The orange and blue bars indicate responding in the Typical group and the Atypical group, respectively. Error bars indicate the standard error of the mean.

1. ***SCRs during Costly US-avoidance acquisition and Generalization test***

*Costly US-avoidance acquisition:*

Participants did not differ in regard to their differential SCR responding to the CSs. The main effect of CS type did not reach significance (*b*CStype= -0.039, *SE* = 0.020, *p* = .051). No other interactions reached significance (smallest *p* = .543). The Bayesian models confirmed the absence of these effects, as 100% of the HDIs depicting the interactions involving CS type, Group, and trait anxiety/intolerance of uncertainty, respectively, fell within ROPE.

*Generalization test*

No interactions involving Stimulus type had reached significance (smallest *p* = .256). Additionally, no interaction involving trait anxiety/intolerance of uncertainty reached significance (smallest *p* = .212).


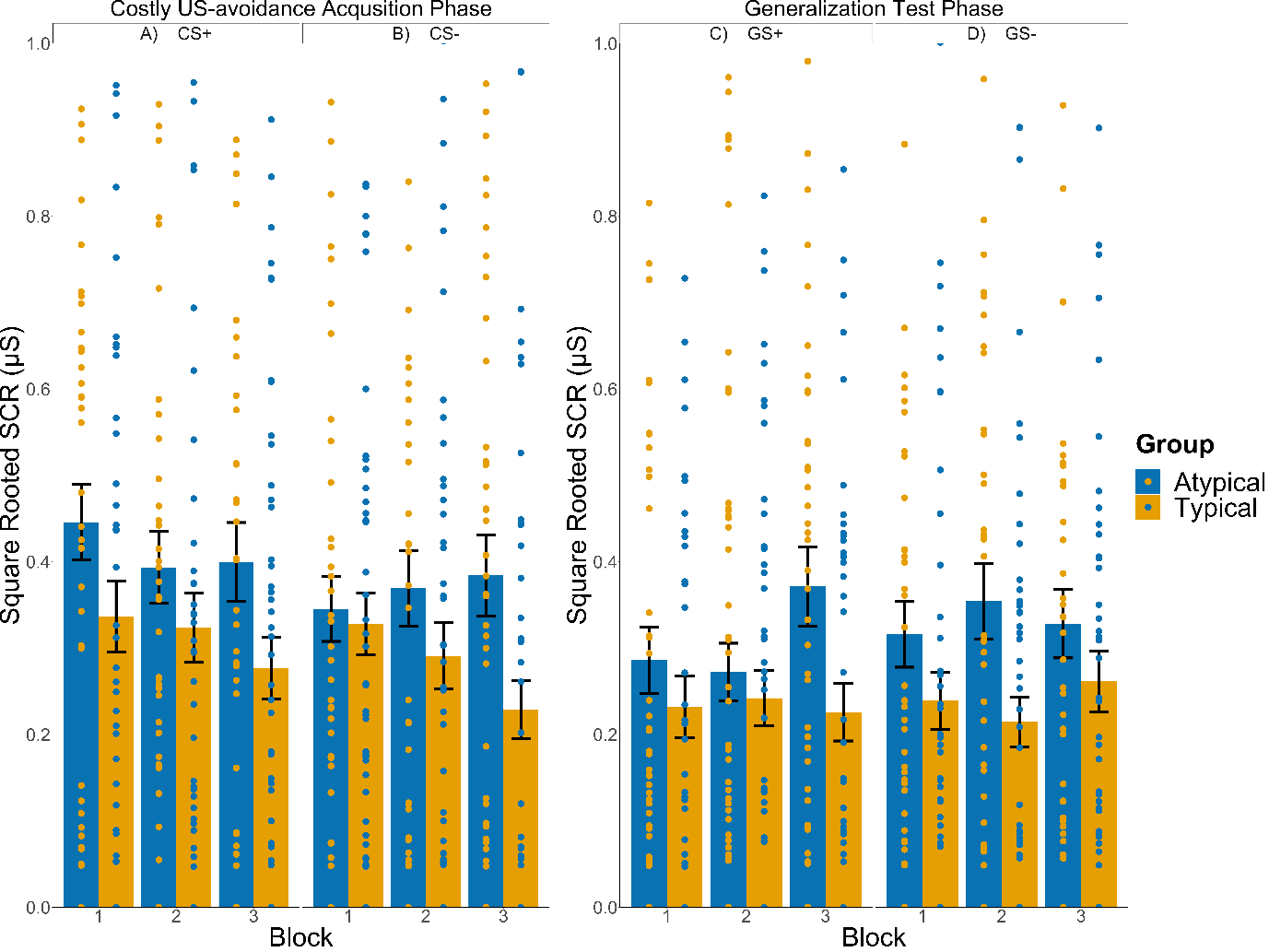


Fig. S4. Left panel: Mean US-expectancy ratings across Costly US-avoidance acquisition blocks for CS+ (A) and CS- (B).

Right Panel: Mean US-expectancy ratings across Generalization test blocks. The orange and blue bars indicate responding in the Typical group and the Atypical group, respectively. Error bars indicate the standard error of the mean.
